# The Arf GTPase-Activating Protein Family Is Exploited by Salmonella enterica Serovar Typhimurium To Invade Nonphagocytic Host Cells

**DOI:** 10.1128/mBio.02253-14

**Published:** 2015-02-10

**Authors:** Anthony C. Davidson, Daniel Humphreys, Andrew B. E. Brooks, Peter J. Hume, Vassilis Koronakis

**Affiliations:** Department of Pathology, Cambridge University, Cambridge, United Kingdom; University of Chicago

## Abstract

To establish intracellular infections, Salmonella bacteria trigger host cell membrane ruffling and invasion by subverting cellular Arf guanine nucleotide exchange factors (GEFs) that activate Arf1 and Arf6 GTPases by promoting GTP binding. A family of cellular Arf GTPase-activating proteins (GAPs) can downregulate Arf signaling by stimulating GTP hydrolysis, but whether they do this during infection is unknown. Here, we uncovered a remarkable role for distinct Arf GAP family members in Salmonella invasion. The Arf6 GAPs ACAP1 and ADAP1 and the Arf1 GAP ASAP1 localized at Salmonella-induced ruffles, which was not the case for the plasma membrane-localized Arf6 GAPs ARAP3 and GIT1 or the Golgi-associated Arf1 GAP1. Surprisingly, we found that loss of ACAP1, ADAP1, or ASAP1 impaired Salmonella invasion, revealing that GAPs cannot be considered mere terminators of cytoskeleton remodeling. Salmonella invasion was restored in Arf GAP-depleted cells by expressing fast-cycling Arf derivatives, demonstrating that Arf GTP/GDP cycles facilitate Salmonella invasion. Consistent with this view, both constitutively active and dominant-negative Arf derivatives that cannot undergo GTP/GDP cycles inhibited invasion. Furthermore, we demonstrated that Arf GEFs and GAPs colocalize at invading Salmonella and collaborate to drive Arf1-dependent pathogen invasion. This study revealed that Salmonella bacteria exploit a remarkable interplay between Arf GEFs and GAPs to direct cycles of Arf GTPase activation and inactivation. These cycles drive Salmonella cytoskeleton remodeling and enable intracellular infections.

## IMPORTANCE

To initiate infections, the Salmonella bacterial pathogen remodels the mammalian actin cytoskeleton and invades host cells by subverting host Arf GEFs that activate Arf1 and Arf6 GTPases. Cellular Arf GAPs deactivate Arf GTPases and negatively regulate cell processes, but whether they target Arfs during infection is unknown. Here, we uncovered an important role for the Arf GAP family in Salmonella invasion. Surprisingly, we found that Arf1 and Arf6 GAPs cooperate with their Arf GEF counterparts to facilitate cycles of Arf GTPase activation and inactivation, which direct pathogen invasion. This report illustrates that GAP proteins promote actin-dependent processes and are not necessarily restricted to negatively regulating cellular signaling. It uncovers a remarkable interplay between Arf GEFs and GAPs that is exploited by Salmonella to establish infection and expands our understanding of Arf GTPase-regulated cytoskeleton remodeling.

## INTRODUCTION

Salmonella enterica is an intracellular bacterial pathogen of worldwide importance causing diseases in animals and humans ranging from acute gastroenteritis to a systemic infection known as typhoid fever (1). To cause disease, Salmonella bacteria invade nonphagocytic intestinal epithelial cells through the action of injected virulence effector proteins that induce cytoskeleton remodeling and membrane ruffling to trigger pathogen macropinocytosis.

Salmonella-induced membrane ruffling requires actin polymerization directed by a cellular machine known as the wave regulatory complex (WRC) (2–4). The WRC is under strict regulation and is governed by a remarkable cooperation between small GTPases Rac1 and Arf1, which directly bind the WRC to mediate its recruitment and activation at the membrane (3, 5, 6). Salmonella bacteria hijack the WRC pathway by elaborate manipulation of small GTPase signaling networks. Small GTPases are activated at the membrane by guanine nucleotide exchange factors (GEFs) that promote GTP binding and are inactivated by GTPase-activating proteins (GAPs), which stimulate GTP hydrolysis to GDP (7, 8). The Salmonella GEF SopE activates Rac (9), which is deactivated in turn by the pathogen GAP SptP (10). Salmonella bacteria encode no known Arf GEF or GAP, so, to mediate WRC-driven uptake, the pathogen must subvert the cellular network of Arf regulatory proteins.

Arf1 is best known for its activities in membrane trafficking at the Golgi membrane, but it is recruited to the plasma membrane by its GEF Arf nucleotide-binding-site opener (ARNO), which activates Arf1 to induce macropinosome formation (3, 11, 12). ARNO is maintained in the cytosol in an auto-inhibited conformation but is recruited and activated at the plasma membrane via Arf6 and acidic phospholipids such as PI(3,4,5)P3 (12, 13). We recently demonstrated that the direct recruitment of ARNO to the membrane by Arf6 triggers WRC-dependent actin polymerization and Salmonella uptake via Arf1 (5). ARNO recruitment to invasion sites was also aided by Arf6 GEFs EFA6 and BRAG2 as well as PI(3,4,5)P3 production via the Salmonella effector SopB (3, 5). Salmonella deactivates Rac1 through SptP, but whether the pathogen deactivates Arf signaling is unknown.

The members of the human Arf GAP family exhibit diverse Arf substrate specificities and can be divided into subfamilies known as ACAP, ADAP, ARAP, ASAP, ArfGAP, and GIT (14) (see Table S1 in the supplemental material). Arf GAP subfamilies GIT, ASAP, ACAP, and ARAP have been implicated in cytoskeleton remodeling, which is mostly attributed to accessory domains found within the complex modular organization of these Arf GAP proteins that determine their localization and scaffold functions (14). For example, the SH3 domain of GIT1 binds the Rho GEF Pix (15), while ACAP is known to interact with integrin β1 (16). Nevertheless, Arf GAP activity itself has also been implicated in cytoskeletal pathways and is thought to downregulate action-based processes (14). For example, the Arf6 GAP activity of ACAP1 blocked formation of actin-rich protrusions dependent on Arf6 (17), whereas the Arf1 GAP activity of ASAP2 impedes dorsal ruffle formation (18, 19). Since Salmonella bacteria orchestrate uptake into host cells through intricate manipulation of the Arf regulatory network, we aimed to address the role of Arf GAPs in the Salmonella invasion process.

## RESULTS

### Specific Arf GAPs localize at sites of Salmonella cytoskeleton remodeling.

**FIG 1:**
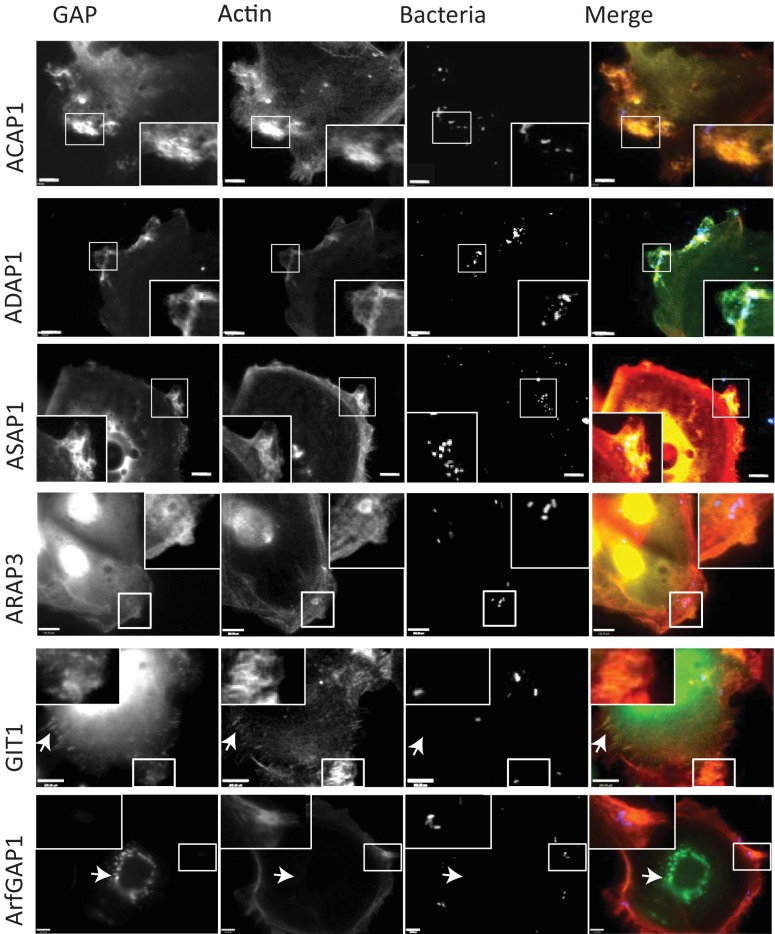
Arf GAP localization at sites of Salmonella cytoskeleton remodeling. Caco2 cells expressing ACAP1, ADAP1, ASAP1, ARAP3, GIT1, or Arf GAP1 fused to fluorescent yellow fluorescent protein (YFP) or cyan fluorescent protein (CFP) (green) as indicated (left) were infected for 15 min with Alexa Fluor 350-labelled (blue) wild-type salmonellae (Bacteria) and stained with Texas Red-phalloidin to visualize the actin cytoskeleton (red). Arrows indicate GIT1-enriched focal adhesions and Golgi localization of ArfGAP1. Insets show magnified areas. Scale bar, 8 µm.

Arf GAPs are known to exhibit divergent localization patterns in mammalian cells. As a first step to resolving whether Arf GAP family members play a role in Salmonella-induced membrane ruffling, we examined their localization during infection of Caco intestinal epithelial cells expressing fluorescent representatives from each subfamily of Arf GAPs (Fig. 1). In each case, Salmonella bacteria were observed triggering extensive remodeling of the cell surface cytoskeleton (actin) that macropinocytosed invading bacteria (magnified insets). Arf GAP1 and GIT1 were not enriched at these pathogen foci and were observed only at the Golgi membrane (Arf GAP1) or focal adhesions (GIT1) (Fig. 1; arrows), which is where they mediate their cellular functions (14). ARAP3 displayed a diffuse distribution and was enriched in the nucleus but not at Salmonella invasion sites (magnified insets). In contrast, ACAP1, ADAP1, and ASAP1 were substantially enriched at Salmonella invasion ruffles (magnified insets). Immunofluorescence showed that endogenous ACAP1, ADAP1, and ASAP1 also localized to Salmonella invasion sites (see Fig. S2 in the supplemental material). Furthermore, ACAP1, ADAP1, and ASAP1 localized with intracellular Salmonella were not observed as confirmed by imaging of Rab5 (see Fig. S2), which is known to colocalize with Salmonella-containing vacuoles (SCVs) following pathogen uptake (20). This shows that the localization of GAPs was restricted to sites of Salmonella cytoskeleton remodeling. As these findings indicate a role for ACAP1, ADAP1, and ASAP1 in Salmonella invasion, they were selected for further investigation.

### Arf GAP activity regulates Salmonella invasion into host cells.

**FIG 2:**
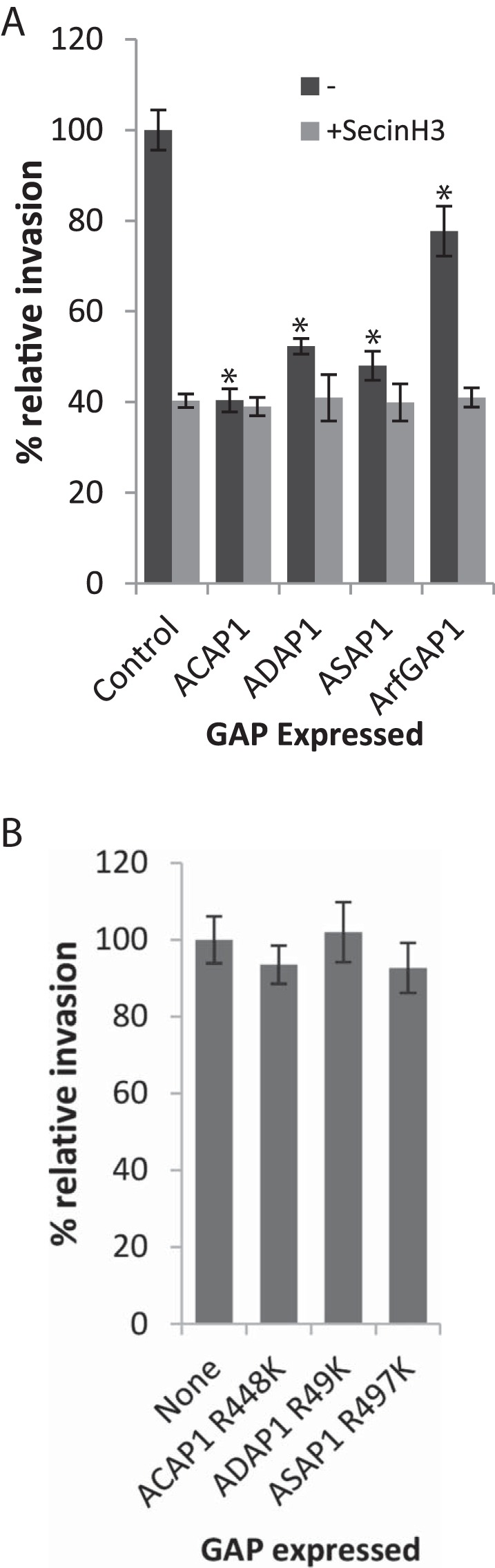
Influence of Arf GAP expression on Salmonella invasion. (A) Salmonella invasion into Caco2 cells expressing recombinant Arf GAPs. Caco2 cells expressing epitope-tagged ACAP1, ADAP1, ASAP1, or Arf GAP1 with or without treatment with SecinH3 were infected (15 min) with Salmonella bacteria carrying pM975 that express GFP inside pathogen-containing vacuoles. Error bars represent ± standard errors of the means (SEM). *, *P* < 0.01. (B) Salmonella invasion into Caco2 cells expressing recombinant Arf GAPs with disabled GAP activity. Caco2 cells expressing an epitope-tagged ACAP1^R448K^, ADAP1^R49K^, or ASAP1^R497K^ GAP mutant were infected (15 min) with Salmonella bacteria carrying pM975 that express GFP inside pathogen-containing vacuoles. Error bars represent ± SEM.

To determine the influence of ACAP1, ADAP1, and ASAP1 on Salmonella remodeling of the cytoskeleton, we examined Salmonella invasion after a 15-min infection of Caco cells individually expressing recombinant hemagglutinin (HA)-tagged Arf GAPs (Fig. 2A). Relative to cells expressing HA vector alone, Salmonella invasion was significantly reduced in cells expressing ACAP1, ADAP1, and ASAP1. The impairment was only modest in cells expressing the Golgi membrane-localized Arf GAP1, which was expressed as a control. The reduction was likely due to the interference of Arf1-dependent formation of CopB1-coated vesicles at the Golgi membrane, which have been shown to promote Salmonella invasion (21). Immunoblotting showed that the differences in levels of Salmonella invasion were not due to disparities in the expression of HA-tagged Arf GAPs (data not shown). These results indicate that increased expression of ACAP1, ADAP1, and ASAP1 (and, to lesser extent, Arf GAP1) inhibited cytoskeleton remodeling at pathogen foci.

Salmonella usurps Arf6 and ARNO to drive Arf1 activation and trigger pathogen-induced ruffling via WRC (3, 5). We have previously shown that inhibiting Arf1 activation at the plasma membrane with a small-molecule inhibitor of ARNO (SecinH3) impairs Salmonella invasion in HeLa cells (3), which was also the case in Caco cells (Fig. 2A; control + SecinH3). Relative to control cells treated with SecinH3, no further reduction in Salmonella uptake was observed when ARNO was inhibited in combination with expression of ACAP1, ADAP1, ASAP1, or ArfGAP1 (Fig. 2A), indicating that, like SecinH3, Arf GAP expression impedes invasion by depleting the pool of active GTP-bound Arf GTPases. Indeed, when Salmonella invasion was examined in cells individually expressing ACAP1, ADAP1, or ASAP1 with disabled GAP activity (R-to-K point mutations), no significant reduction was apparent (Fig. 2B). In conclusion, the GAP activity of ACAP, ADAP, ASAP1, and, to a lesser extent, ArfGAP1 inhibited the Arf1-dependent cytoskeleton remodeling at the plasma membrane responsible for pathogen uptake.

### Arf GAPs regulate distinct Arf GTPases during Salmonella invasion.

**FIG 3:**
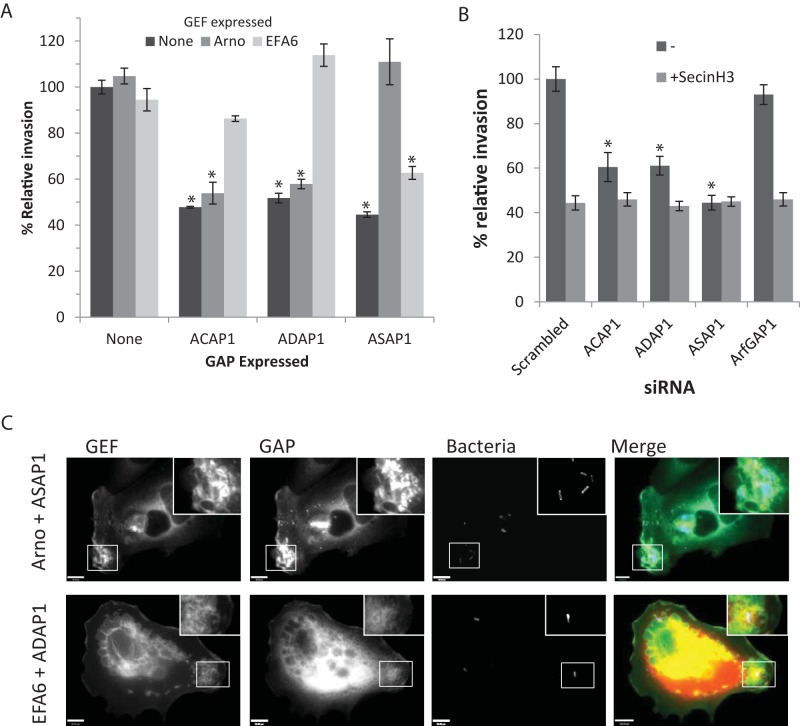
Arf GAP collaboration with Arf GEFs during Salmonella invasion. (A) Salmonella invasion into Caco2 cells expressing recombinant Arf GAPs and GEFs in combination. Caco2 cells expressing epitope-tagged ACAP1, ADAP1, or ASAP1, in combination with ARNO or EFA6, were infected (15 min) with Salmonella bacteria carrying pM975 that express GFP inside pathogen-containing vacuoles. Error bars represent ± SEM. *, *P* < 0.01. (B) Salmonella invasion into Caco2 cells transfected with siRNA targeting Arf GAPs in the presence or absence of SecinH3. Caco2 cells were transfected with siRNAs targeting ACAP1, ADAP1, ASAP1, or ArfGAP1, in the presence or absence of SecinH3, and were infected (15 min) with Salmonella bacteria carrying pM975 that express GFP inside pathogen-containing vacuoles. Error bars represent ± SEM. *, *P* < 0.01. Gene knockdown was quantified by qRT-PCR (data not shown). (C) Caco2 cells coexpressing CFP-ARNO with ASAP2-YFP and dsRed-EFA6 with CFP-ADAP1. Cells were infected for 5 min with Alexa Fluor 350-labelled (blue) wild-type salmonellae (Bacteria) and fixed with 4% paraformaldehyde (PFA). Scale bar, 8 µm.

As Arf6 and Arf1 play distinct roles in WRC-dependent actin assembly, we sought to determine which Arfs are specifically deactivated by ACAP1, ADAP1, or ASAP1. The GEF EFA6 activates Arf6, which recruits and activates ARNO at the plasma membrane (5, 11, 12). ARNO then triggers WRC-dependent actin polymerization by activating Arf1 (3, 5). Consistent with this, EFA6 and ARNO are known to increase the pools of GTP-bound Arf6 and Arf1, respectively (11). This being the case, we reasoned that expression of recombinant EFA6 would counteract Arf6 GAPs whereas ARNO would offset Arf1 GAPs, thereby revealing how ACAP1, ADAP1, and ASAP1 regulate pathogen macropinocytosis (Fig. 3A). In control cells, expression of EFA6 had no significant influence on invasion, which was also the case when EFA6 was coexpressed with ASAP1, as cytoskeleton remodeling remained impaired due to GAP activity. In contrast, EFA6 restored invasion to 80% of the level seen with the control in ACAP-expressing cells and invasion was completely restored, indeed, enhanced, to ~115% in ADAP-expressing cells. Consistent with the deactivation of Arf6, Salmonella invasion remained impaired when the Arf1 Gef ARNO was expressed in ACAP1- and ADAP1-expressing cells. In contrast, Salmonella invasion into ASAP1-expressing cells was restored to control levels by ARNO expression. These findings show that Arf GAPs regulate distinct Arf GTPases and therefore regulate distinct steps in the cellular signaling underlying Salmonella invasion. This is consistent with the view that ACAP1 and ADAP1 deactivate Arf6 whereas ASAP1 targets Arf1 (14).

### Arf GAPs ACAP1, ADAP1, and ASAP1 facilitate ARNO-dependent Salmonella invasion.

Arf1 and Arf6 are known to trigger actin polymerization in their GTP-bound conformation by binding downstream signaling proteins WRC and ARNO, respectively (3, 5). In support of this view, promoting Arf inactivation through expression of Arf GAPs inhibited pathogen invasion (Fig. 2A). We reasoned that loss of Arf GAP activity would increase the pool of GTP-bound Arfs and augment Salmonella invasion. We thus examined Salmonella invasion in Caco cells depleted of the Arf6 GAPs ACAP1 and ADAP1 or the Arf1 GAP ASAP1 by small interfering RNA (siRNA) transfection (Fig. 3B), whose knockdown was confirmed by quantitative reverse transcription-PCR (qRT-PCR) (see Fig. S3 in the supplemental material). Surprisingly, Salmonella invasion was significantly impaired in ADAP1-, ACAP1-, and ASAP1-depleted cells. These phenotypes contrasted with those observed in cells depleted of Arf GAP1, which had no effect on Salmonella invasion, and were consistent with its absence from pathogen foci (Fig. 1). Similarly, depletion of the Arf GAPs ARAP and GIT1 (see Fig. S3) also had no effect on invasion (data not shown).

These findings show that, like Arf GEFs, members of the Arf GAP family facilitate invasion. We speculated that Arf GEFs and GAPs might collaborate during pathogen macropinocytosis, which is known to hinge on the action of ARNO. Indeed, when invasion was examined in the presence of SecinH3, no further reduction was observed in ADAP- and ASAP1-depleted cells (Fig. 3B), suggesting that they promote ARNO-driven cytoskeleton remodeling. Furthermore, when we studied the localization of Arf GAPs in Arf GEF-expressing cells, we found that ADAP1 and ASAP1 colocalized with their GEF counterparts EFA6 and ARNO at sites of Salmonella-induced cytoskeleton remodeling (Fig. 3C).

### Cycles of Arf GTPase activation and deactivation facilitate Salmonella invasion.

**FIG 4:**
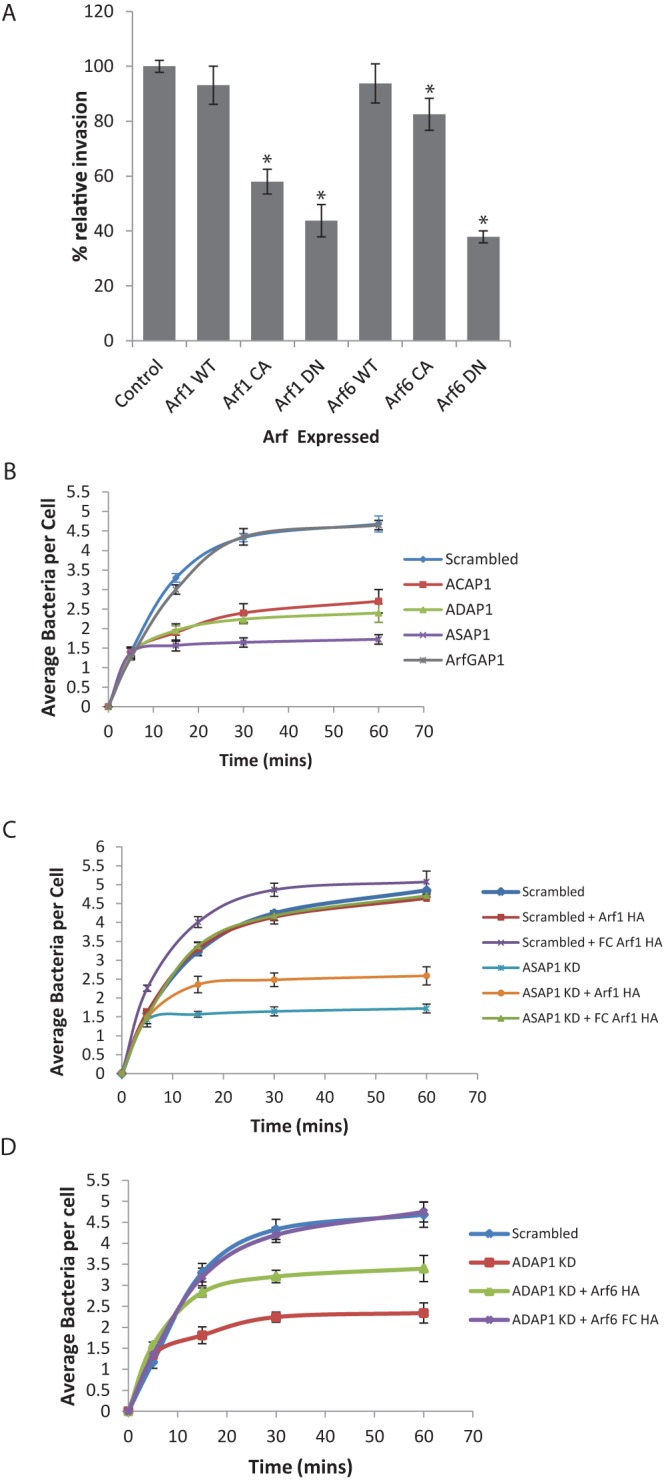
Influence of Arf GDP/GTP cycles on Salmonella invasion. (A) Salmonella invasion into Caco2 cells expressing recombinant Arf proteins. Caco2 cells expressing epitope-tagged Arf1 WT, Arf1 CA, Arf1 DN, Arf6 WT, Arf6 CA, or Arf6 DN were infected (15 min) with Salmonella bacteria carrying pM975 that express GFP inside pathogen-containing vacuoles. Error bars represent ± SEM. *, *P* < 0.01. (B) Time course of Salmonella invasion into Caco2 cells transfected with siRNAs targeting Arf GAPs. Caco2 cells transfected with control (scrambled) ACAP1, ADAP1, ASAP1, or ArfGAP1 siRNAs were infected for the indicated times with Salmonella bacteria carrying pM975 that express GFP inside pathogen-containing vacuoles. Error bars represent ± SEM. *, *P* < 0.01. Gene knockdown was quantified by qRT-PCR (see Fig. S3 in the supplemental material). (C) Salmonella invasion into ASAP1-depleted Caco2 cells expressing recombinant Arf1 variants. Caco2 cells transfected with control (scrambled) or ASAP1 siRNAs (knockdown [KD]) were subsequently transfected with empty vector or HA-tagged wild-type Arf1 (Arf1 HA) or fast-cycling Arf1 (FC Arf1 HA) and then infected for the indicated times with Salmonella bacteria carrying pM975 that express GFP inside pathogen-containing vacuoles. Error bars represent ± SEM. *, *P* < 0.01. Gene knockdown was quantified by qRT-PCR (see Fig. S3). (D) Salmonella invasion into ADAP1-depleted Caco2 cells expressing recombinant Arf6 variants. Caco2 cells transfected with control (scrambled) or ADAP1 siRNAs (knockdown [KD]) were subsequently transfected with empty vector or HA-tagged wild-type Arf6 (Arf6 HA) or fast-cycling Arf6 (Arf6 FC HA) and then infected for the indicated times with Salmonella bacteria carrying pM975 that express GFP inside pathogen-containing vacuoles. Error bars represent ± SEM. *, *P* < 0.01. Gene knockdown was quantified by qRT-PCR (see Fig. S3).

We reasoned that, if Arf GEFs and GAPs collaborate, cycles of Arf activation (GTP binding) and deactivation (GTP hydrolysis) rather than sustained activation of Arfs may be key to Salmonella cytoskeleton remodeling. To test this hypothesis, we examined Salmonella invasion in Caco cells expressing wild-type (WT), constitutively active (CA), and dominant-negative (DN) Arf1 or Arf6 derivatives (Fig. 4A). Constitutively active Arfs are locked in a GTP-bound conformation, while dominant-negative isoforms are in GDP-bound or nucleotide-free inactive conformations and sequester endogenous Arf GEFs. The results of Salmonella invasion into cells expressing Arf1-WT and Arf6-WT were equivalent to those seen with the control. As expected, Arf1-DN and Arf6-DN, which are locked in an inactive conformation, impaired invasion (Fig. 4A). Remarkably, expression of Arf1-CA also impaired invasion, which was equivalent to that seen for DN variants, while Arf6-CA expression resulted in a modest but statistically significant reduction in invasion. These results indicate that cycles of activation and deactivation of Arf1 and, to a lesser extent, Arf6 facilitate pathogen invasion.

We speculated that, without the ability to deactivate Arfs, the plasma membrane pool of Arf GTPases would be quickly exhausted and impede successive rounds of Salmonella invasion. To examine this possibility, we first investigated the influence of impaired Arf deactivation on Salmonella invasion over 60 min in cells depleted of ACAP1, ADAP1, ASAP1, or Arf GAP1 (Fig. 4B). At 5 min, equivalent numbers of intracellular bacteria were apparent in each case, demonstrating an efficient initial burst of Salmonella invasion. In control (scrambled siRNA-depleted) and Arf GAP1-depleted cells, the numbers of intracellular bacteria continued to increase at similar rates during the 60 min. In contrast, no significant increase in the number of internalized bacteria was evident from 5 min in cells depleted of ASAP1, and no increase after 15 min was observed when ACAP1 or ADAP1 was depleted. These data show that impeding the deactivation of Arf1 and Arf6 at the plasma membrane abrogates the ability of Salmonella to drive invasion for more than 5 min and 15 min, respectively.

It was apparent that depletion of ASAP1 had a greater inhibitory effect than depletion of ACAP1 or ADAP1 (Fig. 4B). This was not due to redundancy between the Arf6 GAPs since depletion of both ACAP1 and ADAP1 by double siRNA transfection resulted in no further reduction in Salmonella invasion relative to the results seen with cells depleted of ACAP1 or ADAP1 alone (see Fig. S4B in the supplemental material). This suggests that deactivation of Arf1 is more critical to Salmonella invasion than deactivation of Arf6. Consistent with this view, expression of constitutively active Arf1 had a greater inhibitory effect on invasion than expression of constitutively active Arf6 (Fig. 4A).

Our results support the view that Arf GDP/GTP cycles are dispensable for a single bacterium to invade host cells but that cycling is required for the internalization of multiple bacteria infecting the same cell. To test this hypothesis, we restored Arf cycling in Arf GAP-depleted cells by engineering fast-cycling (FC) derivatives of Arf1 and Arf6 that rapidly bind and hydrolyze GTP themselves and thus mimic the dynamic cycles of activation and deactivation stimulated by GEFs and GAPs (22). These experiments also functionally controlled for off-target effects following siRNA transfection. In (scrambled) control cells, expression of either wild-type or fast-cycling Arf1 and Arf6 derivatives had little effect on Salmonella invasion (Fig. 4C and D). ASAP1 depletion impaired Salmonella invasion over 60 min, but this was restored to control levels by expression of Arf1-FC (Fig. 4C). In contrast, expressing Arf1-WT could not fully restore invasion in ASAP1-depleted cells and resulted in an increase in the number of bacteria that was only incremental. This indicates that the additional Arf1 was also quickly deactivated and depleted from the plasma membrane, preventing further rounds of invasion from occurring. Similarly, Arf6-FC but not Arf-WT was able to restore invasion in ADAP1-depleted cells (Fig. 4D). Furthermore, expression of Arf1-FC could not restore Salmonella invasion in ADAP1-depleted cells and Arf6-FC was unable to restore pathogen uptake in ASAP1-depleted cells (data not shown), reaffirming that the Arf1 GAP ASAP1 and Arf6 GAP ADAP1 cooperate with their GEF counterparts ARNO and EFA6 in Salmonella cytoskeleton remodeling.

Interestingly, Arf1-FC was incapable of restoring Salmonella invasion into cells where both ARNO and ASAP1 were inhibited by a combination of specific siRNAs (see Fig. S4B in the supplemental material). This shows that ARNO was still required to recruit Arf1-FC to the plasma membrane as previously shown for Arf1-WT during Salmonella infection (3). In contrast, Arf6 has an intrinsic affinity for the plasma membrane (7) and Arf6-FC was capable of partially restoring invasion in cells depleted of both EFA6 and ADAP1 (see Fig. S4C).

### ASAP1 releases Arf1 signaling complexes from the membrane.

**FIG 5:**
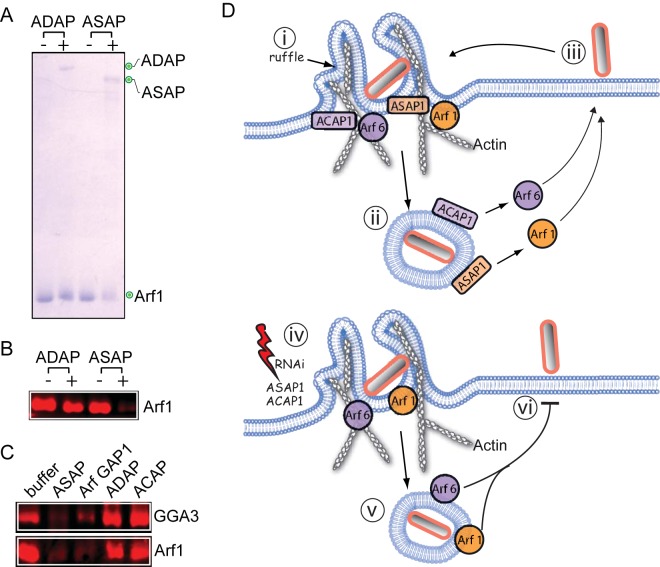
Arf GAP disassembly of Arf1-protein complexes at the membrane. (A) Arf1 interaction with the membrane in the presence of GAPs of Arf6 and Arf1. Silica beads coated with phospholipid bilayers were anchored with active GTP-bound myristoylated Arf1 and then incubated in buffer with or without the GST-tagged Arf6 GAP ADAP1 or the Arf1 GAP ASAP1. Beads were washed, and the remaining membrane-associated proteins were extracted and analyzed by SDS-PAGE and Coomassie blue staining. (B) Samples from the experiment whose results are presented in panel A were immunoblotted with antibodies against Arf1. (C) Interaction of Arf1-GGA3 complexes with the membrane in the presence of Arf GAPs. Membranes anchored with active GTP-bound myristoylated Arf1 complexed with the GAT domain of GGA3 were incubated with Arf1 GAPs (ASAP1 and Arf GAP1) or Arf6 GAPs (ACAP1 and ADAP1). Beads were washed, and the remaining membrane-associated proteins were extracted and analyzed by the use of SDS-PAGE and immunoblotting antibodies against Arf1, His (Arf GAP1 and ACAP1), and GST (ASAP1 and ADAP1). (D) Model: a single Salmonella bacterium generates a membrane ruffle and invades host cells in a manner dependent on Arf1 and Arf6 (i), both of which are present on macropinosomes. The colocalization of the Arf GAPs ACAP1, ADAP1, and ASAP1 with Arf1 and Arf6 on macropinosomes promotes recycling (to the plasma membrane) of Arf1 and Arf6 (ii), which are exploited by extracellular Salmonella bacteria to facilitate additional invasion events into the same cell (iii). A single invasion event is possible in ACAP1, ADAP1, or ASAP1 RNAi cells (iv), and Arf1 and Arf6 are present on macropinosomes (v), but, due to loss of deactivation, Arf recycling and that of their cellular effectors is impaired, which inhibits new Salmonella invasion events (vi).

How does Arf GAP activity promote cytoskeleton remodeling? It is known that Arf1 binds membranes only when in its active GTP-bound form (23). We reasoned that Arf1 deactivation would release the GTPase and its cognate cellular effectors from the membrane to supply a ready pool of signaling components for other cell processes. To test this hypothesis, we first assessed the ability of ASAP1 to release Arf1 from the membrane. Purified myristoylated Arf1 was loaded with GTP and anchored to silica microspheres coated in a phospholipid bilayer before incubation in buffer alone (−) or in buffer containing the ADAP1 or ASAP1 (Fig. 5A). When incubated with buffer or the Arf6 GAP ADAP, Arf1 remained at the membrane, but Arf1 was clearly released following incubation with ASAP1. Immunoblotting confirmed that ASAP1 released Arf1 from the membrane, which resulted in an ~75% reduction in the membrane-anchored Arf1 level relative to the level seen with the control (Fig. 5B). To investigate whether ASAP1 could disassemble Arf1-membrane signaling assemblies, we formed a complex between membrane-anchored Arf1-GTP and the GAT domain of its known cellular effector GGA3 (GGA3^GAT^) before incubation with Arf GAPs was performed (Fig. 5C). Arf1 and GGA3^GAT^ remained associated at the membrane when incubated with buffer or the Arf6 GAPs ADAP and ACAP. In contrast, ASAP1 released the Arf1-GGA3^GAT^ complex from the membrane. This phenomenon was not specific to ASAP1 and could be triggered by any Arf1 GAP as demonstrated by the use of Golgi protein Arf GAP1 but not Arf6 GAPs ACAP1 and ADAP1.

## DISCUSSION

The activity of small GTPases is controlled by a repertoire of cellular GEFs and GAPs that exhibit discrete substrate specificities and subcellular distributions to enable actin polymerization at precise membrane locations. We previously discovered that WAVE complex activation requires coincident binding by Arf1 and Rac1, which directs Salmonella-induced ruffling (3, 6). Arf1 is normally found at the Golgi membrane but is recruited to Salmonella invasion sites via an Arf6 network that controls ARNO, the Arf1 GEF (3). Host cells encode an array of Arf GAPs that would likely modulate Arf GTPases at Salmonella foci, but this issue had not been addressed.

Arf GAPs are known to display diverse subcellular localization patterns ranging from the Golgi membrane to the plasma membrane (14). Given the extraordinary ability of bacterial pathogens to manipulate cellular signaling components, it seemed possible that Salmonella might exclude Arf GAPs from pathogen foci in order to sustain activation of Arfs and facilitate Salmonella invasion via the WAVE complex. We show that this is not the case. In fact, Arf GAPs ACAP1, ADAP1, and ASAP1 were considerably enriched at sites of Salmonella cytoskeleton remodeling. To our knowledge, this is the first time that colocalized host GAPs, Arf or otherwise, have been observed at bacterial infection foci. This localization was specific, as ARAP3 was found at the plasma membrane but was not substantially enriched at invasion sites, and neither were Arf GAP1 and GIT1.

What is the role of GAPs at Salmonella invasion sites? GAPs are thought to terminate signaling pathways by stimulating GTP hydrolysis in small GTPases. Indeed, Salmonella exploits this paradigm by delivering SptP, a virulence effector with Rac1 GAP activity that returns the actin cytoskeleton to a resting state following pathogen internalization (10). Consistent with this view, enhanced expression of recombinant ACAP1, ADAP1, or ASAP1, which would deplete the pool of active Arf, inhibited invasion. Our findings are in line with other studies that have overexpressed GAPs to examine their role in cytoskeleton remodeling. For example, expression of ASAP1 and ACAP1 was recently shown to inhibit the formation of ventricle actin structures (19). However, loss of ACAP1, ADAP1, or ASAP1 also impaired Salmonella invasion, showing that Arf deactivation is needed for efficient pathogen macropinocytosis. This establishes that Arf GAPs cannot be considered mere terminators of signaling pathways and that they can actually promote actin polymerization. Indeed, the Arf GAP activity of ARAP2 has been suggested to promote formation of filopodia (24) whereas GIT2 is required for the generation of podosomes (25).

How does deactivation of Arf1 and Arf6 enable pathogen invasion? We provide evidence that Arf GDP/GTP cycles are key to cytoskeleton rearrangements rather than sustained Arf GTPase activation. Salmonella invasion was restored in cells depleted of Arf1 or Arf6 GAPs by expressing fast-cycling Arf1 or Arf6. Furthermore, Salmonella invasion was inhibited by expressing Arf variants locked in a constitutively active conformation. In support of our hypothesis, fast-cycling Arf6 has been shown to potentiate action-based protrusions and membrane trafficking, which were inhibited by constitutively active Arf6 (22, 26). Furthermore, we showed that Arf1 and Arf6 GAPs colocalized with their GEF counterparts at Salmonella invasion foci, where they work in synergy to drive pathogen uptake.

How does deactivation aid Salmonella cytoskeleton remodeling? Arf1 associates with membranes in a GTP-dependent manner and colocalizes with the WAVE complex on Salmonella macropinosomes, which undergo trafficking to a perinuclear position (3). To drive successive rounds of Salmonella invasion, the pool of Arf1 at the plasma membrane must be replenished, especially as the majority of Arf1 is found at the Golgi membrane. Deactivation of Arf1 by its GAPs would release the GTPase and its binding partners from the membrane (e.g., macropinosomes), which could be targeted to pathogen invasion sites through GEFs to permit further rounds of Salmonella uptake. In support of this view, we demonstrated that Arf1 GAPs released a complex of Arf1 and GGA3 from the membrane (Fig. 5C). This is an established paradigm at the Golgi membrane, where Arf1 generates secretory vesicles by recruiting the COPI coat protein (27). Following vesicle formation, Arf1-COP1-membrane complexes are disassembled by Arf GAP1 deactivation of Arf1 to enable further rounds of vesicle formation. It is therefore likely that the same Arf1 mechanism is in operation at the plasma membrane, which would enable the cell to recycle cytoskeletal regulators. Similarly, deactivation of Arf6 by ACAP1 or ADAP1 may release ARNO from macropinosomes to increase the pool of ARNO for plasma membrane activation of Arf1. Furthermore, constitutively active Arf6 is known to accumulate on intracellular endosomes at a perinuclear position (28) and Arf6 deactivation is likely necessary to return Arf6 to the plasma membrane.

Interestingly, we found that deactivation of Arf1 was more crucial than deactivation of Arf6 (Fig. 4A and B). Arf6 is abundant at the plasma membrane, and yet there are only low levels of Arf1, which needs to be replenished in order to drive the generation of new macropinosomes. We showed that this is achieved only following Arf1 deactivation by ASAP1. Multiple factors are known to recruit ARNO (7) and likely compensate for misregulation of Arf6. For example, we showed that the PIP3 phosphoinositide promotes localization of ARNO to Salmonella invasion sites (3).

A number of bacterial pathogens, including Salmonella (SptP), Legionella (LepB), Yersinia (YopE), and Pseudomonas (ExoS) spp., encode functional mimics of mammalian GAP proteins (29). Bacterial exploitation of host GAPs appears to be a much more unusual phenomenon. Interestingly, all pathogen-encoded GAPs are thought to downregulate cell processes. For example, the Salmonella Rac1 GAP—SptP—returns the host cell cytoskeleton to a resting state following pathogen uptake whereas Yersinia YopE inhibits bacterial internalization (10, 30). Remarkably, our report illuminates the finding that bacteria exploit host GAPs to promote cytoskeleton remodeling and pathogen invasion. Consistent with this, Listeria monocytogenes is known to exploit mammalian Arf GAP activity of ARAP2 to enter host cells through bacterial surface proteins InlA and InlB (31). Our study demonstrated that Salmonella exploits two sets of host Arf GAPs: those that control Arf1 and those that control Arf6 (Fig. 5D). This remarkable interplay between the cellular GEFs and GAPs of Arf1 and Arf6 enables Salmonella bacteria to drive macropinocytosis into the host cells where the intracellular pathogen causes disease.

## MATERIALS AND METHODS

### Plasmids, recombinant proteins, and antibodies.

DNA primers (Table 1) were used to generate plasmids (Table 2) by Invitrogen by the use of Gateway methodology. Point mutations were introduced into target genes by site-directed mutagenesis using the instructions of the manufacturer (Agilent Technologies). The following plasmids were kindly provided to us: pM975 (Wolf-Dietrich Hardt) and pET-arf1 and pBB131 encoding the Arf family N-myristoyltransferase (Martin Spiess). Glutathione *S*-transferase (GST)- and His-tagged proteins were expressed in Escherichia coli Rosetta (Novagen) at 16°C before affinity purification was performed (3). Antibodies were purchased from Abcam (actin, ACAP1, ADAP1, and ASAP1), Sigma (FLAG), Pierce (GST), and Covance (HA).

**TABLE 1:** DNA primers

Primer	PCR product(s)	Primer sequence
Gateway vectors		
ACAP 268–519 GW F	ACAP1 GAP and PH	GGGGACAAGTTTGTACAAAAAAGCAGGCTCCATGGAAGGACATCTCTTCA
ACAP 268–519 GW R		GGGACCACTTTGTACAAGAAAGCTGGGTCTCACAGGAACTTCTTCTCCAC
ACAP1 Gw F	Full-length ACAP1	GGGGACAAGTTTGTACAAAAAAGCAGGCTCCATGACGGTCAAGCTGGATT
ACAP1 Gw R		GGGACCACTTTGTACAAGAAAGCTGGGTCATGCAGCGTGTGGAGGTCATG
ADAP1 Gw F	Full-length ADAP1	GGGGACAAGTTTGTACAAAAAAGCAGGCTCCATGGCCAAGGAGCGGCGCA
ADAP1 Gw R		GGGACCACTTTGTACAAGAAAGCTGGGTCCTAAGGTTTATGCTTGAAGTG
ARAP3 289–1084 GW F	GAP, PH, and Rho GAP ARAP3	GGGGACAAGTTTGTACAAAAAAGCAGGCTCCATGAGTGGCTGGCTAGACA
ARAP3 289–1084 GW R		GGGACCACTTTGTACAAGAAAGCTGGGTCCTAGTAGCCATCAATGAGCTC
ArfGAP1 1–136 GW F	GAP domain ArfGAP1	GGGGACAAGTTTGTACAAAAAAGCAGGCTCCATGGCCAGCCCAAGAACCA
ArfGAP1 1–136 GW R		GGGACCACTTTGTACAAGAAAGCTGGGTCTCATGGGGTCCAGTTCTGGGC
ASAP1 316–665 GW F	GAP and PH ASAP1	GGGGACAAGTTTGTACAAAAAAGCAGGCTCCATGAAGAAGGGGTACCTGC
ASAP1 316–665 GW R		GGGACCACTTTGTACAAGAAAGCTGGGTCGATCATAGGTTTTCAAGGAAG
		
Mutants		
ACAP1 R448Q F	GAP mutation ACAP1	TGTTCCGGCATCCACCAGAGCCTTGGTGTTCAC
ACAP1 R448Q R		GTGAACACCAAGGCTCTGGTGGATGCCGGAACA
Arf1 T161A F	Fast-cycling Arf1 mutation	CAGGCCACCTGCGCCGCCAGCGGCGACGGGCTC
Arf1 T161A R		GAGCCCGTCGCCGCTGGCGGCGCAGGTGGCCTG
Arf6 T175A F	Fast-cycling Arf6 mutation	CAGCCCTCCTGTGCCGCCTCAGGGGACGGACTC
Arf6 T175A R		GAGTCCGTCCCCTGAGGCGGCACAGGAGGGCTG
ASAP1 R497K F	GAP mutation ASAP1	TGTTCTGGCATCCATAAGGAAATGGGGGTTCAT
ASAP1 R497K R		ATGAACCCCCATTTCCTTATGGATGCCAGAACA

**TABLE 2:** Expression plasmids

Plasmid	Source
Mammalian expression plasmids	
pDest ACAP1-YFP	This study
pDest eCFP ADAP1	This study
pDest eCFP ArfGAP1 1–136	This study
pDest ASAP1-YFP 316–665	This study
pDest ASAP2-YFP 1–539	This study
pDest eCFP ARAP3 289–1084	This study
pEGFP Git1	Addgene 15226
pDest eCFP Arno	Humphreys et al. 2012 (3)
pDest eCFP EFA6A 1–645	Humphreys et al. 2013 (5)
pcDNA FLAG ACAP1	This study
pcDNA nHA ACAP1 R448K	This study
pcDNA nHA ADAP1	This study
pcDNA nHA ADAP1 R49K	This study
pcDNA nHA ArfGAP1 1–136	This study
pcDNA nHA ASAP1 316–665	This study
pcDNA nHA ASAP1 316–665 R497K	This study
pcDNA nHA ARAP3 289–1084	This study
pcDNA nHA Arno	This study
pcDNA nHA EFA6A 1–645	Humphreys et al. 2013 (5)
pcDNA cHA Arf1	Humphreys et al. 2012 (3)
pcDNA cHA Arf1 Q71L	This study
pcDNA cHA Arf1 T31N	This study
pcDNA cHA Arf1 T161A	This study
pcDNA cHA Arf6	This study
pcDNA cHA Arf6 Q67L	This study
pcDNA cHA Arf6 T27N	This study
pcDNA cHA Arf6 T157A	This study
pmRFP-Rab5	Addgene 14437
Bacterial expression plasmids	
pGEX2T ADAP1	This study
pGEX2T ASAP1 316–665	This study
pbEN-SPB-SET2a ACAP1 268–519	This study
pHis ArfGAP1 1–136	This study
pGEX-GGA3-GAT	Humphreys et al. 2012 (3)

### Bacterial strains and infection of Caco2 cells.

Wild-type S. enterica serovar Typhimurium SL1344 (gift from Jean Guard-Petter) was used in all experiments. For fluorescence microscopy, bacteria were washed with phosphate-buffered saline conjugated to Alexa Fluor 350 carboxylic acid succinimidyl ester (15 min, 37°C), washed in Tris (pH 7.4)-buffered saline, and then used to infect Caco2 cells (multiplicity of infection [MOI] of 50). For fluorescence microscopy, fixed infected cells were stained with Alexa Fluor 594-phalloidin to visualize actin. To quantify invasion, Salmonella bacteria (carrying pM975) that express green fluorescent protein (GFP) via the SPI2 promoter once the bacteria are within Salmonella-containing vacuoles (SCVs) (32) were used to infect (15 min) Caco2 cells. The number of fluorescent bacteria per cell (~400 cells per experiment) was then counted using microscopy. When appropriate, Caco2 cells were incubated with 25 µM SecinH3 (Merck). Immunofluorescence microscopy was performed and images were assembled as previously described (3). All experiments were performed at least three times. Geometric means were calculated, and significance was determined by Student’s *t* test or one-way analysis of variance (ANOVA) followed by a *post hoc* Dunnett’s comparison. A *P* of <0.01 was considered significant.

### Mammalian cell culture and transfections.

Mammalian Caco2 cells were routinely cultured in complete growth media consisting of minimal essential medium (MEM) supplemented with 10% (vol/vol) fetal bovine serum (FBS), 2 mM l-glutamine, 200 µg/ml^−1^ 0.1 mM nonessential amino acids, streptomycin, and 100 U·ml^−1^ penicillin (37°C, 5% CO_2_). Transient transfection of Caco2 cells by microporation was performed using a Neon transfection system according to the instructions of the manufacturer (Invitrogen). For RNA interference (RNAi) analysis, small interfering RNA (siRNA) from Qiagen and Dharmacon (Table 3) was transfected into Caco2 cells with Oligofectamine transfection reagent (Invitrogen) according to the instructions of the manufacturer. EFA6A and ARNO siRNA have been previously described (3, 5). The transfection mixture was replaced after 24 h with complete growth medium, and cells were cultured for 72 h in total. RNA interference (RNAi) efficiency was determined by Express One-Step SYBR GreenER qRT-PCR according to the instructions of the manufacturer (Invitrogen) with actin used as a relative control in each case.

**TABLE 3:** siRNAs

Gene product	siRNA	Target sequence	Catalog no.
ASAP1	Hs_DDEF1_5	CAGACTCGCCCACATCACCAA	SI04135145
	Hs_DDEF1_6	GAGGCGAGTGAAGACCATTTA	SI04181800
ArfGAP1	Hs_ArfGAP1_7	CGCGGCCCTCTTTAGGGATAA	SI03194814
	Hs_ArfGAP1_8	AGCGGTCTGGACCACTTCCAA	SI04142656
ACAP1	Hs_CENTB1_1	CACCGTGAGCCTGAACCACAA	SI00343273
	Hs_CENTB1_2	ACGGGCCAGCAACGCATTTAA	SI00343280
ARAP3	Hs_CENTD3_4	CTGGAGTAATGAGATAGTACA	SI00343434
	Hs_CENTD3_5	GAGACGCTTTGTGCAGTTCAA	SI04148403
ASAP2	Hs_DDEF2_3	CACGTTCACGTTGAATATGAA	SI00360619
	Hs_DDEF2_6	TAGGAAGTCGCAGGCAACCAA	SIO4151784
ADAP1	Hs_CENTA1_1	CCGGAAGTTTGTGCTGACAGA	SI00092771
	Hs_CENTA1_2	GAGGCGCACTTCAAGCATAAA	SI00092764
GIT1	Hs_GIT1_5	CAGCCTTGACTTATCCGAaTT	SI02224467
	Hs_GIT1_6	GCCGCTGAGGATGTCCCGAAA	SI02622340

### Reconstituting Arf1 interaction with Arf GAPs and GGA3.

Preparation of phospholipid-coated beads and anchoring of myristoylated GTP-loaded Arf1 to the beads has been previously described in detail (6). Arf1-anchored phospholipid-coated beads were incubated with Arf GAPs in HKSM (10 mM HEPES [pH 7.4], 100 mM KCl, 1 mM MgCl) for 10 min before the beads were washed and membrane-bound proteins extracted with SDS-urea. To form Arf1-GGA3^GAT^, Arf1-anchored phospholipid-coated beads were incubated with GGA3^GAT^, washed, and then incubated with Arf GAPs.

## SUPPLEMENTAL MATERIAL

Figure S1  Localization of endogenous Arf GAPs at Salmonella invasion sites. Caco2 cells were infected for 15 min with Alexa Fluor 350-labelled (blue) wild-type Salmonella (bacteria) and stained with ACAP1, ADAP1, or ASAP1 antibodies to visualize endogenous Arf GAPs (green). Scale bar, 10 µm. Download Figure S1, PDF file, 2.5 MB

Figure S2  Localization of Arf GAPs following Salmonella uptake. Arf GAP localization on Salmonella-containing vacuoles (SCVs) as marked by Rab5 is shown. Caco2 cells expressing red fluorescent protein (RFP)-Rab5 together with ACAP1-YFP, CFP-ADAP1, or ASAP1-YFP were infected for 10 min with Alexa Fluor 350-labelled (blue) wild-type salmonellae (bacteria) and then incubated for a further 10 min in the presence of gentamicin to prevent further invasion and determine Arf GAP localization on SCVs. Scale bar, 10 µm. Download Figure S2, PDF file, 2.2 MB

Figure S3  RNAi-mediated knockdown of Arf GAPs. Results of qRT-PCR quantification of mRNA levels of indicated Arf GAPs relative to control levels (100% in each case) after 72-h incubation with siRNAs are shown. Download Figure S3, PDF file, 0.6 MB

Figure S4  Influence of Arf GDP/GTP cycles on Salmonella invasion in the absence of GEF and GAP activity. (A) Salmonella invasion into Caco2 cells transfected with control (scrambled) ACAP1 or with ADAP1 siRNA alone or into cells transfected with both ACAP1 and ADAP1 siRNA. (B) Salmonella invasion into Caco2 cells transfected with control (scrambled) ASAP1 siRNA alone or in combination with ARNO siRNA with or without expression of recombinant Arf1-FC. (C) Salmonella invasion into Caco2 cells transfected with control (scrambled) ADAP1 with or without EFA6 siRNA and subsequently transfected with empty vector (−) or HA-tagged fast-cycling Arf6-FC. In the experiments whose results are shown in panels A, B, and C, cells were infected for 15 min with Salmonella bacteria carrying pM975 that express GFP inside pathogen-containing vacuoles. Error bars represent ± SEM. *, *P* < 0.01 (relative to scrambled control) (ANOVA; see Materials and Methods). Asterisks above brackets indicate a *P* value of *P* < 0.01 (for the difference in relative values determined by the Student’s *t* test). Download Figure S4, PDF file, 0.6 MB

Table S1  Arf GAP family. The table lists the 6 Arf GAP subfamilies and their target Arf substrates. Table S1, DOC file, 0.03 MB.
