# Supplementary figures and images for: The Arf GTPase-Activating Protein Family Is Exploited by Salmonella enterica Serovar Typhimurium To Invade Nonphagocytic Host Cells
Source: mBio. 2015 Feb 10;6(1):e02253-14. doi: 10.1128/mBio.02253-14 (PMC4337568; doi:10.1128/mBio.02253-14)

Fig.S1 - Localisation of endogenous Arf GAPs localisation at Salmonella invasion sites

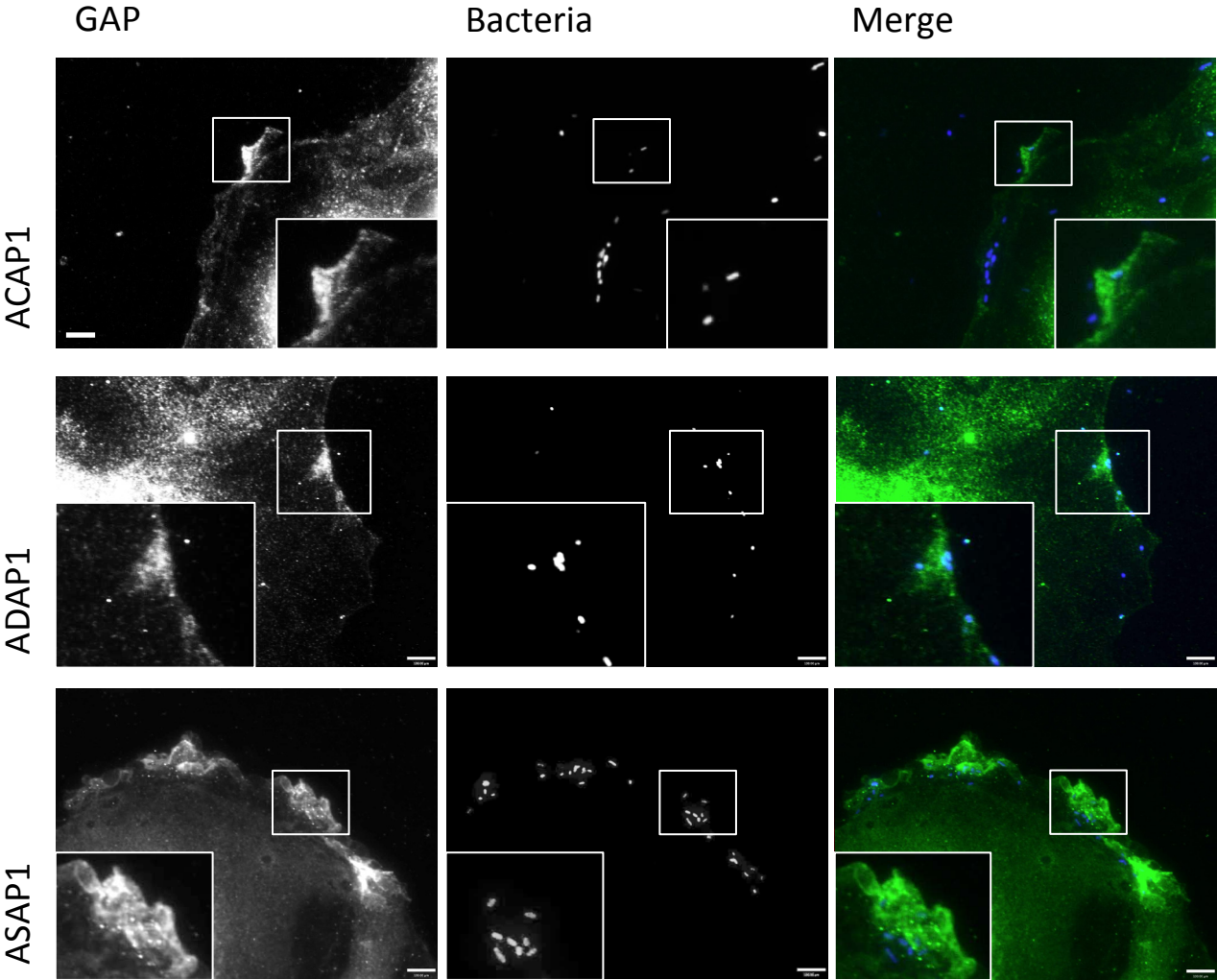

Supplement: Figure S1 — Localization of endogenous Arf GAPs at Salmonella invasion sites. Caco2 cells were infected for 15 min with Alexa Fluor 350-labelled (blue) wild-type Salmonella (bacteria) and stained with ACAP1, ADAP1, or ASAP1 antibodies to visualize endogenous Arf GAPs (green). Scale bar, 10 µm. Download [file mbo001152179sf1.pdf]

Fig.S2 - Localisation of Arf GAPs following Salmonella uptake

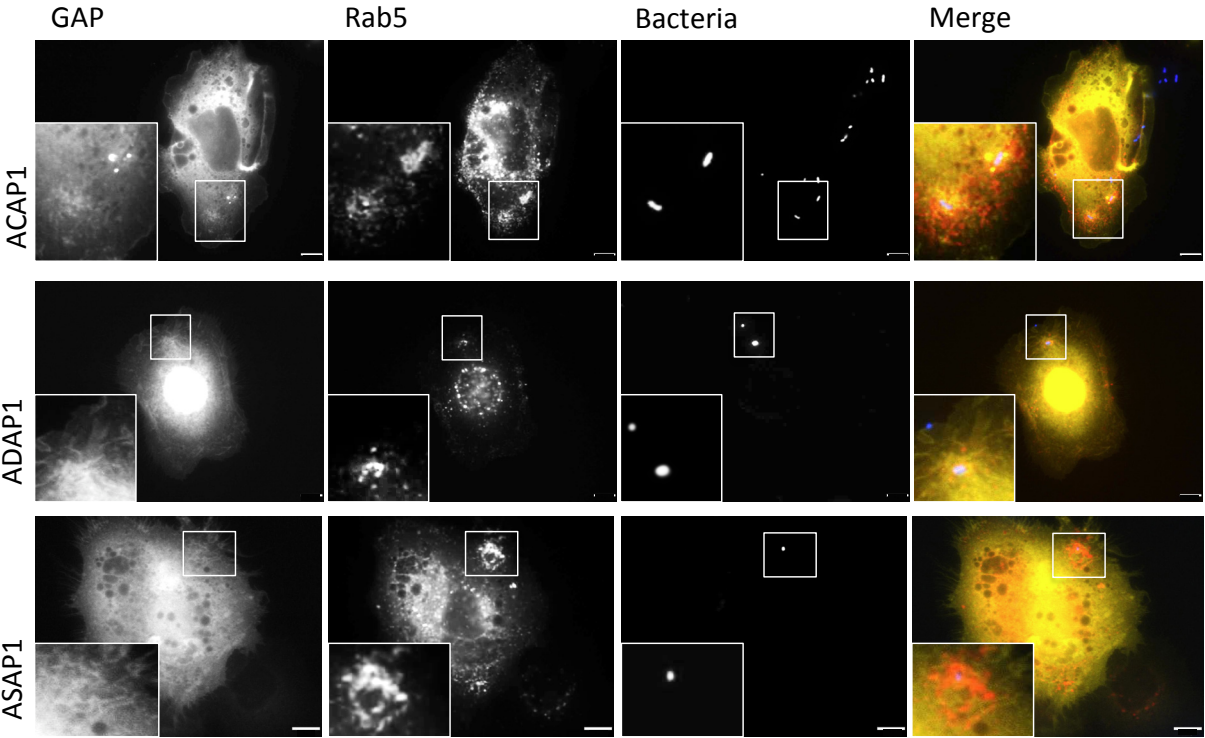

Supplement: Figure S2 — Localization of Arf GAPs following Salmonella uptake. Arf GAP localization on Salmonella-containing vacuoles (SCVs) as marked by Rab5 is shown. Caco2 cells expressing red fluorescent protein (RFP)-Rab5 together with ACAP1-YFP, CFP-ADAP1, or ASAP1-YFP were infected for 10 min with Alexa Fluor 350-labelled (blue) wild-type salmonellae (bacteria) and then incubated for a further 10 min in the presence of gentamicin to prevent further invasion and determine Arf GAP localization on SCVs. Scale bar, 10 µm. Download [file mbo001152179sf2.pdf]

Fig.S3 - RNAi-mediated knockdown of Arf GAPs

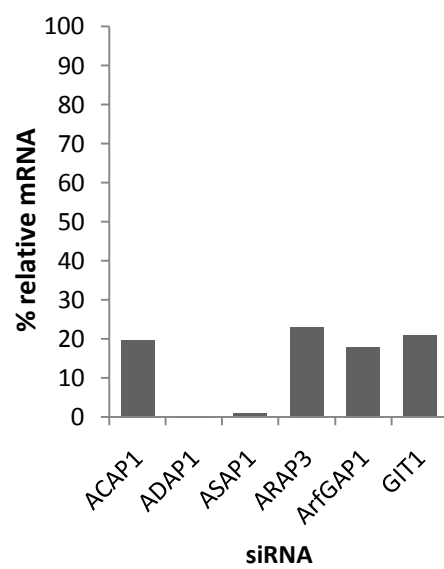

Supplement: Figure S3 — RNAi-mediated knockdown of Arf GAPs. Results of qRT-PCR quantification of mRNA levels of indicated Arf GAPs relative to control levels (100% in each case) after 72-h incubation with siRNAs are shown. Download [file mbo001152179sf3.pdf]
