# Supplementary material for: The Arf GTPase-Activating Protein Family Is Exploited by Salmonella enterica Serovar Typhimurium To Invade Nonphagocytic Host Cells
Source: mBio. 2015 Feb 10;6(1):e02253-14. doi: 10.1128/mBio.02253-14 (PMC4337568; doi:10.1128/mBio.02253-14)

Fig.S4 - Influence of Arf GDP/GTP cycles on Salmonella invasion in the absence of GEF and GAP activity

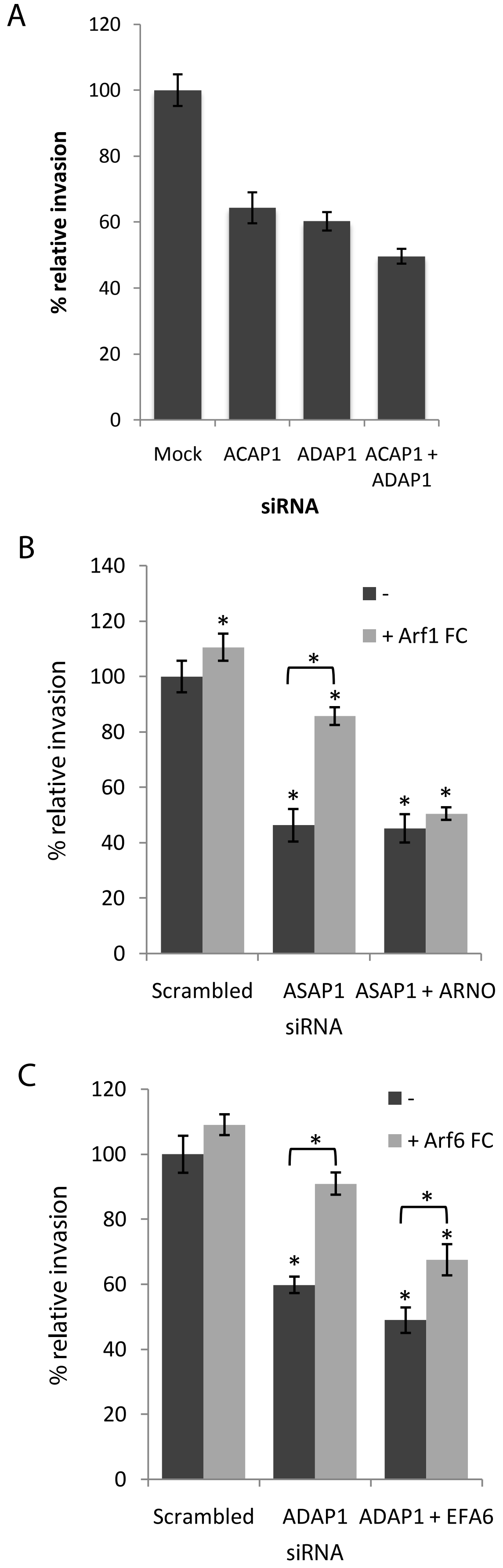

Supplement: Figure S4 — Influence of Arf GDP/GTP cycles on Salmonella invasion in the absence of GEF and GAP activity. (A) Salmonella invasion into Caco2 cells transfected with control (scrambled) ACAP1 or with ADAP1 siRNA alone or into cells transfected with both ACAP1 and ADAP1 siRNA. (B) Salmonella invasion into Caco2 cells transfected with control (scrambled) ASAP1 siRNA alone or in combination with ARNO siRNA with or without expression of recombinant Arf1-FC. (C) Salmonella invasion into Caco2 cells transfected with control (scrambled) ADAP1 with or without EFA6 siRNA and subsequently transfected with empty vector (−) or HA-tagged fast-cycling Arf6-FC. In the experiments whose results are shown in panels A, B, and C, cells were infected for 15 min with Salmonella bacteria carrying pM975 that express GFP inside pathogen-containing vacuoles. Error bars represent ± SEM. *, P < 0.01 (relative to scrambled control) (ANOVA; see Materials and Methods). Asterisks above brackets indicate a P value of P < 0.01 (for the difference in relative values determined by the Student’s t test). Download [file mbo001152179sf4.pdf]
