# Supplementary material for: The Arf GTPase-Activating Protein Family Is Exploited by Salmonella enterica Serovar Typhimurium To Invade Nonphagocytic Host Cells
Source: mBio. 2015 Feb 10;6(1):e02253-14. doi: 10.1128/mBio.02253-14 (PMC4337568; doi:10.1128/mBio.02253-14)
Supplement: Table S1 — Arf GAP family. The table lists the 6 Arf GAP subfamilies and their target Arf substrates. [file mbo001152179st1.doc]

**Table S1: Arf GAP family.**

| **GAP family** | **substrate** |
| --- | --- |
| ACAP1-3 | Arf6 |
| ADAP1-2 | Arf6 |
| ARAP1-3 | Arf1,5,6 |
| ASAP1-3 | Arf1,5 |
| ArfGAP1-3 | Arf1 |
| GIT1-2 | Arf6 |
